# Complete response to temozolomide in chronic lymphocytic leukemia

**DOI:** 10.1002/ccr3.978

**Published:** 2017-05-31

**Authors:** Archana Rao, Nisha Ramani, Robert Stoppacher, Thomas Coyle

**Affiliations:** ^1^ Hematology Oncology Associates of CNY5008 Brittonfield ParkwayEast Syracuse13057New York; ^2^ Department of PathologySUNY Upstate Medical University750 East Adams streetSyracuse13210New York; ^3^ Department of Benign HematologyTriHealth Cancer Institute10506 Montgomery RdCincinnati45242Ohio

**Keywords:** Chemotherapy, CNS tumors, lymphoma

## Abstract

We report a case of incidentally diagnosed chronic lymphocytic leukemia (CLL) in a patient with glioblastoma, which responded completely following standard treatment of the glioblastoma with temozolomide and cranial irradiation. The patient remained without an evidence of CLL until his death from recurrent glioblastoma. Further study of temozolomide for the treatment of CLL is indicated.

We report a patient with glioblastoma multiforme who was treated with surgery followed by radiation therapy and temozolomide. He incidentally had Binet stage A chronic lymphocytic leukemia (CLL) at diagnosis, which responded completely to this treatment. Chlorambucil has been the standard alkylating agent used for CLL for many years. However, the complete and overall response rates for chlorambucil monotherapy range from only 0–7% and 31–72%, respectively 1, 2. Chemo‐immunotherapy with fludarabine, cyclophosphamide, and rituximab yields much higher rates of complete responses that are sometimes durable, but not thought to be curative and is associated with significant toxicity, especially in the elderly 3. The observation of a complete response of CLL to temozolomide is therefore interesting.

The patient was a previously healthy 60‐year‐old man who presented with confusion, headache, and right‐sided visual disturbance and was found to have a mass in the left cerebral hemisphere that was completely resected and found to be glioblastoma. He was treated with postoperative irradiation with concomitant temozolomide at a dose of 75 mg/m^2^ orally daily for 42 days and then with temozolomide at a dose of 150–200 mg/m^2^ daily for 5 days monthly for eight cycles as described by Stupp et al. 4. His glioblastoma recurred. Salvage treatments with bevacizumab and irinotecan and, subsequently, lomustine were given, but he succumbed from progressive glioblastoma.

At presentation, a complete blood count showed white blood cell count of 42.5 × 10^9^/L, an absolute lymphocyte count of 29.7 × 10^9^/L, a hemoglobin of 12.1 g/dL, and a platelet count of 159 × 10^9^/L. Peripheral blood cytometry showed a monoclonal kappa‐restricted B‐cell population co‐expressing CD19, CD20, CD23, CD5, and FMC‐7 consistent with chronic lymphocytic leukemia. FISH analysis showed an interstitial deletion of chromosome 13q (q14–q14.3). He had no symptoms or abnormalities on physical examination referable to the CLL. An abdominal CT showed a rounded heterogeneous lesion in the spleen measuring 7.5 cm. There was no adenopathy. No specific treatment of the CLL was indicated.

At the conclusion of temozolomide therapy, the patient had a WBC of 6.4 × 10^9^/L, a hemoglobin of 12.7 g/dL, a platelet count of 158 × 10^9^/L, and an absolute lymphocyte count of 0.5 × 10^9^/L. Peripheral blood flow cytometry showed no evidence of CLL. The splenic lesion did not change on CT. A postmortem examination performed 7 months after the conclusion of temozolomide treatment showed no evidence of CLL in the blood, bone marrow, spleen, or any other tissue. The splenic lesion was shown to be a histiocytic sarcoma, clonally unrelated to the CLL.

This patient also received corticosteroids for cerebral edema and external beam radiotherapy, both of which may contribute to lymphocytotoxicity. However, both have been used as adjunctive treatments for CLL and complete responses are not seen from these treatments. The complete response seen in this case is much more likely to be due to temozolomide.

Temozolomide has several properties, suggesting it may be useful in the treatment of CLL. It is generally well tolerated with a dose limiting toxicity of myelosuppression, occurring in 7% of patients treated with on the Stupp trial 4. Elderly patients are reported to tolerate temozolomide well 5. Lymphopenia with attendant risks for Pneumocystis Jiroveci pneumonia and herpes zoster, however, is temozolomide's most characteristic toxicity 6. Temozolomide's effect on depletion of T cells and NK cells has been extensively documented. It also has also been shown to deplete B cells with a significant decrease in both the percentage and absolute number of CD19 + B lymphocytes 7. Furthermore, temozolomide has been employed as a lympho‐depleting agent to enhance vaccine‐driven immune responses in murine models of melanoma and in pilot clinical trials in glioblastoma patients 8.

Temozolomide is indicated for the treatment of glioblastoma and anaplastic astrocytoma in adults. It also finds unlabeled use in mycosis fungoides, Ewings sarcoma, metastatic melanoma, soft tissue sarcoma, neuroendocrine tumors, and primary CNS lymphoma. Neither a PubMed search nor discussion with the drug manufacturer revealed reports for the treatment of CLL with temozolomide. This serendipitously observed complete response, coupled with its lymphocytotoxic activity, ease of oral administration and generally well‐tolerated safety profile even in the elderly provide rationale for the formal study of temozolomide's activity in CLL.

## Authorship

AR: first author who performed literature review and wrote the manuscript. NR: pathology resident involved in conducting the gross autopsy and microscopic examination. RS: Pathology attending physician who conducted the gross autopsy and drafted the autopsy report. TC: Senior Author who treated the patient and co‐wrote the manuscript.

## Conflict of Interest

None declared.
